# The National Health Insurance Scheme (NHIS) in the Dormaa Municipality, Ghana: Why Some Residents Remain Uninsured?

**DOI:** 10.5539/gjhs.v6n3p82

**Published:** 2014-02-21

**Authors:** Thompson Amo

**Affiliations:** 1 Public Health Management, Graduate School of Asia, Pacific Studies, Ritsumeikan Asia Pacific University, Beppu, Japan

**Keywords:** NHIS, subscription, Dormaa Municipal, Ghana

## Abstract

The paper presents a quantitative investigation on the National Health Insurance Scheme (NHIS) in the Dormaa Municipality, Ghana: Why some residents remain uninsured? Since its implementation a little over a decade now. The aim is to identify the obstacles of enrolment by the public, which would enable policy direction, to ensure that all residents are registered with the scheme. A descriptive and cross-sectional study was conducted between May and July, 2013. Both purposive and simple random sampling techniques were used to select 210 respondents and data obtained through self-administered, and face-to-face interviews guided by structured questionnaire. Chi square (χ^2^) test of independence was adopted to show the association between socioeconomic and demographic features as well as membership. Findings from the research suggest that residents’ decision to enrol is significantly associated with gender, education, number of children, place of residence, employment and income. It was also observed that membership is highly affected by premium level. The discussion of the findings and recommendations offered, if incorporated into the policy guideline of NHIS, could maintain, and at the same time increase enrolment level. This would guarantee quality and affordable basic health care protection for the good people of Ghana.

## 1. Introduction

Health is very essential to every nation and individual. Living a healthy life enables individuals to exhibit his/her full potential to create more opportunities for themself and their country. The amount of money involved in accessing health care in time of need is always an obstacle to most of the citizenry, especially the poor. Household surveys by world health organization member countries have shown that, about 150 million people are faced with financial difficulties every year because of direct health care cost. There are a lot of instances where people lose their lives because they cannot make an upfront payment before seeking Medicare. Universal Health Coverage (UHC) has thus become one of the prime targets for health care transformation in several countries, and a significant objective of the World Health Organization (WHO). Governments all over the world have great concern on health matters of the people. In May 2005, the fifty-eighth World Health Assembly adopted a resolution urging member states to consider using alternative mechanisms of resource mobilization; including social health insurance in order to bridge the gap between the rich and the poor in accessing health care. Good health is the key to the sustainability of economic and social development, thereby lessening poverty in society. The access to the required health care needs is essential and vital to maintain and improve health care. Simultaneously, people needed to be protected to avoid being pushed into poverty due to the costs of seeking health care. Evidence from the World Bank (WB) in 2006 estimated that, 28.5% of Ghanaians are living with a dollar per day. Despite this, 27.0% of household income is spent on health care (WHO, 2010).

### 1.1 Health Insurance in Ghana

Ghana mostly relies on international support for human and capital development, faced with a slow pace of economic advancement and institutional limitations. The common phenomenon facing developing countries explains the need to undertake a robust measure to design a means to finance health care is very important. The National Health Insurance act (ACT 650) backed by legislative instrument (LI 1809) was passed in 2004 with the sole responsibility of ensuring that access to quality health care is free for all without any difficulties through the establishment of mutual health insurance schemes in all the districts in the country (Yevutsey & Aikins, 2010). The Act established the National Health Insurance Authority (NHIA) to regulate, facilitate and coordinate the activities of all the district base health insurance schemes across the country. The NHIS covers primary health care services which constitute about 95% of frequently reported cases in the health care institutions in the country comprising the charge of drugs acknowledged in the NHIS drug list. Outpatient and inpatient services such as eye care services, maternity care, oral health services, surgical and gynaecological operations and emergency care are covered under the scheme.

Premiums are determined according to the poverty indicators in every district, and only make it mandatory for people between the ages of 18 and 69 years to pay yearly subscription fees. Persons 70 years and above and also Children below 18 years whose parents are beneficiary are also registered free of charge. The national health insurance regulation also provides that people lacking visible financial source, no permanent residence, not living with someone who is employed with permanent residence or not having a persistent and consistent source of income from others is considered as indigent, and relieved from premium payment. Pregnant women also enjoyed the same benefit as they are exempted from all financial obligations of NHIS.

It is expected that, the introduction of NHIS must have a significant impact on health indicators. The life expectancy of the total population is 63.5 years and the chance of dying between the ages of 15 and 60 per 1,000 of population is 234.5 (WHO, 2013). The 2011 estimate of WHO on out of pocket expenditure as a percentage of private expenditure on health is 66.3%. The 2011 annual report of NHIS indicates a total active membership of 8.2 million, which represent 33% of the population. But the question that still remains unanswered is; why for almost a decade now, a significant number of people are unwilling to register? Could this be attributed to an institutional problem or the general public? This study aims at identifying the specific obstacles that affect residents’ decision to enrol in the scheme and offer credible remedies based on available facts to cure this mischief using Dormaa Municipality as a reference point.

## 2. Methods

A descriptive and cross-sectional study design using a qualitative method was adopted in this paper. Three-stage selection criteria were used to select respondents for the study. In the initial stage, purposive sampling was used to group communities into urban and rural communities, and only two urban cities were selected. In the second stage, a simple random sampling was adopted to select 10 additional rural communities. The last stage involves the selection of respondents using simple random sampling based on the population size of each community on the assumption that information would be obtained from both rural and urban residents in order to draw a fair and balanced conclusion. In all, 210 respondents from the Dormaa Municipality in the Brong Ahafo Region of Ghana were included in the study. Data was obtained through face-to-face interviews (with respondents whose educational background could not assist them to complete the questionnaire), and self-administered for the educated ones; guided by well-structured questionnaires. The questionnaires were divided into three main fragments. The first part looked at socioeconomic and demographic features such as age, gender, education, number of children, place of residence, employment and income. The second and the last sections respectively focused on the reasons for not joining the scheme and suggestions to ensure total coverage. Data was collected between May and July, 2013.

### 2.1 Data Analysis Plan

Statistical Package for Social Sciences (SPSS version 18.0) was used in analysing the data for easy understanding of the result. A bivariate analysis such as chi square (χ^2^) test of independence was also engaged to show the association between the dependent and the independent variables.

## 3. Results

TABLE 1 above presents the socioeconomic and demographic characteristics of the sampled population. There are 126 (60%) subscription and 84 (40%) non-subscription. The mean age of the whole respondents is 43.5 with a significance value of 0.124. There are 44.3% male and 55.7% female respondents which show a significant figure of 0.002. Females have higher membership of 36.7% compared to male subscription of 23.3%. The results also show that the majority of respondents have education up to Middle/Junior High School level (40%). There is a significant association between education and insurance subscription (χ^2^ = 20.387, df = 5, P = 0.001). Married persons constitute the higher number of respondents (48.6%) but the single and widowed have higher subscription levels (62.5% each). The analysis shows no significant difference between insurance subscription and marital status (χ^2^ = 0.482, df = 3, P = 0.923). There is a significant association between the number of children and the demand of health insurance (χ^2^ = 22.017, df = 4, P = 0.000). People who live in the rural communities have a higher subscription level (39.5%) over those in the urban centres (20.5%), with a significant association between place of residence and purchasing of health insurance (χ^2^ = 17.043, df = 1, P = 0.000). The results further show that there is a significant difference between insurance and employment. The sample involves more farmers (33.3%) and at the same time higher subscription level (20.0%). The income level of the respondents revealed a significant association between the amount received and the ability to demand for health insurance (χ^2^ = 37.59, df = 5, P = 0.000). The income level of the sampled population show that 67.0% of the respondents earn monthly income between 0-200 (GH ¢200 = USD $105.3); which is very low.

**Table 1:** The impact of socioeconomic and demographic features over enrolment

Socioeconomic and Demographic Data	Groupings	Current Subscribers		Total (%)	Pearson Chi Square value	P-Value
		Yes (%) n=126	No (%) n=84			
Age	18-30	37 (56.9)	28 (43.1)	65 (30.9)
	31-43	46 (67.6)	22 (32.4)	68 (32.4)
	44-56	19 (46.3)	22 (53.7)	41 (19.5)
	57-69	24 (66.7)	12 (33.3)	36 (17.2)
	Total	126	84	210	5.767	0.124
Gender	Male	49 (52.7)	44 (47.3)	93 (44.3)
	Female	77 (65.8)	40 (34.2)	117 (55.7)
	Total	126	84	210
Education Level	No Education	12 (100)	0	12 (6.2)
	Non Formal	0	8 (100)	8 (4.1)
	Primary School	17 (60.7)	11 (39.3)	28 (14.4)
	Middle/JHS	50 (64.1)	28 (35.9)	78 (40.0)
	Tech/Comm/SHS/‘O’ Level	19 (52.8)	17 (47.2)	36 (18.5)
	Tertiary	25 (75.8)	8 (24.4)	33 (16.8)
	Total	123	72	195	20.387	0.001
Marital Status	Single	40 (62.5)	28 (37.5)	68 (32.4)
	Married	62 (60.8)	40 (39.2)	102 (48.6)
	Divorced	4 (50.0)	4 (50.0)	8 (3.8)
	Widowed	20 (62.5)	12 (37.5)	32 (15.2)
	Total	126	84	210	0.482	0.923
Number of Children/Dependents	0	37 (56.9)	28 (43.1)	65 (31.0)
	1	12 (100)	0	12 (5.6)
	2	33 (76.7)	9 (23.3)	43 (20.5)
	3	19 (43.3)	27 (58.7)	46 (21.9)
	≥4	24 (54.5)	20 (45.5)	44 (21.0)
	Total	125	84	210	22.017	0.000
Place of Residence	Rural	83 (72.8)	31 (27.2)	114 (54.3)
	Urban	43 (44.8)	53 (55.2)	96(45.7)
	Total	126	84	210	17.043	0.000
Employment Status	Apprenticeship	20 (100)	0	20 (9.5)
	Farmer	42 (60.0)	28 (40.0)	70 (33.3)
	Salary Worker	21 (65.6)	11 (34.4)	32 (15.3)
	Self Employed	13 (54.2)	11 (45.8)	24 (11.4)
	Student	6 (37.5)	10 (62.5)	16 (7.6)
	Trader	24 (60.0)	16 (40.0)	40 (19.1)
	Unemployed	0	8 (100)	8 (3.8)
	Total	126	84	210	29.47	0.000
Income level	<100	37 (74.0)	13 (26.0)	50 (27.5)
	100-200	46 (63.9)	26 (36.1)	72 (39.5)
	201-300	1 (8.3)	11 (91.7)	12 (6.6)
	301-400	8 (100)	0	8 (4.4)
	401-500	10 (83.3)	2 (16.7)	12 (6.6)
	>500	8 (28.6)	20 (71.4)	28 (15.4)
	Total	110	72	182	37.59	0.000

Chi-Square test is statistically significant at P< 0.05 level (Asymp. Sig. (2-sided)

Note: 1 USD $ = GH ¢1.90 as of March, 2013 by Bank of Ghana

### 3.1 Reasons for Not Joining the NHIS by Respondents

**Figure 1:**
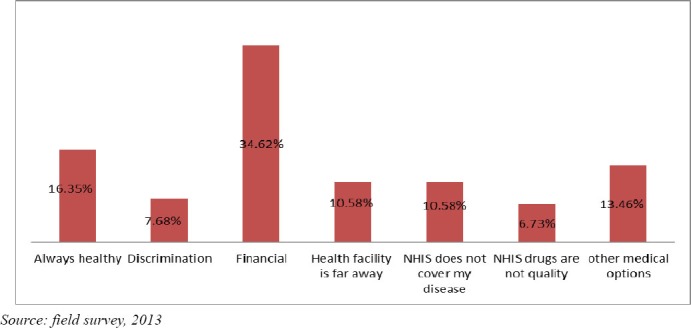
Reasons for non-membership of the NHIS by respondents

Figure 1 illustrates the respondents’ reasons why they remained uninsured at the time of the study. In all, 40.0% of the respondents authenticated their non-subscription of the insurance. The research sought to know their reasons for their action since the main objective of the research is to identify why some residents remain uninsured. This would permit evidence based information for policy transformation, and strategies in securing full endorsement by the people.

A lot of reasons were assigned by respondents for their non-participation in the scheme, but the most pressing among them are: financial (34.62%), always healthy (16.35%), other medical options (13.46%), the NHIS benefit package does not cover my health problem (10.58%), health facility is far away from my community (10.58%), discrimination at the point of service (7.69%) and NHIS drugs are substandard (6.73%).

### 3.2 Suggestions to Ensure Total Coverage by Respondents

**Figure 2:**
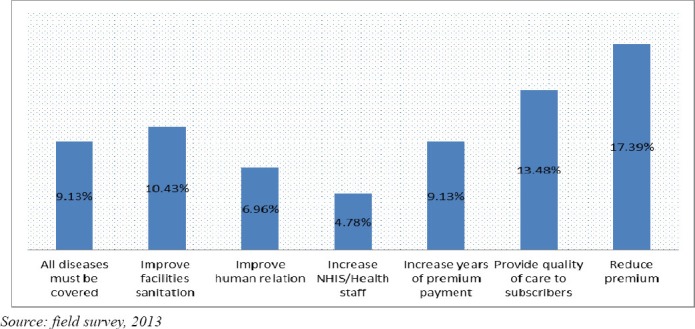
The respondents’ suggestions to achieve universal coverage

The respondents’ were asked to suggest what they think in their opinion could be done to ensure total coverage. Several suggestions among others were stated, but the most pressing ones are illustrated in figure 2.

Figure 2 shows that 17.39% of the respondents suggested a reduction of the premiums, 13.48% believed that the quality of health care must be improved, 10.43% suggested an improvement in facility sanitation, 9.13% each represent people who says that all diseases must be covered and an increase in years of premium payment, improve human relation was suggested by 6.96% of the respondents, and lastly, increase NHIS/Health staff represents 4.78% of the respondents. Other measures such as refurbishment of modern equipment’s of all facilities (2.73%), Speed up card process (2.54%), instant registration at regular interval (2.42%), reimburse of transport fares to patients (2.34%), avoid discrimination at the point of service (1.97%), NHIS card must be accepted by all facilities (1.93%), and NHIS must ensure quality standards for prescriptions (1.87%); among others were mentioned as a key to ensure total coverage.

## 4. Discussion

The Respondents were selected from different backgrounds to ensure accuracy of results and the actual situation pertaining in the municipality. A total of 210 respondents were selected across the length and breadth of the municipality for the study. The statistics showed that 60.0% are members of NHIS, and over 40.0% are non-subscribers. The socioeconomic characteristics such as age, gender, education, place of residence, marital status, number of children/dependents, employment and income was analysed and linked to insurance subscription. The findings suggest that young adults (18-43 years) have a higher subscription (39.5%) over those within the ages of 44-69 years (20.4%). It is proven that the age of the people has no significant influence to the individual decision to enrol in the insurance scheme. This contradicts the finding by Gobah et al. (2011), Kirigia et al. (2005) and Grossman (1972) that age has a major effect on health insurance ownership. The high subscription of the young adults could be linked to the youthful nature of the population. It is estimated that 0-14 years formed 38.4%, 15-64 years (57.7%) and 65 and above is 3.9% of the population (WB, 2011).

The gender composition of respondents in relation to their subscription was determined to evaluate their involvement. Findings revealed that 44.3% and 55.7% represent a male-female respondent which is closely in line with the general population and housing census in 2010 which tag women population to 51.0%, as against men (49.0%), of the municipal population. The test shows that, females are more involved in the scheme than men and in general, gender is statistically significant on enrolment. This again contradicts (Gobah et al., 2011) finding that sex has no significant influence on the people’s decision to demand health care.

Findings on the education level show a significant influence on the individual quest to buy health insurance. Their results depict that as people moves towards getting a higher education, their demand for health insurance increases. The various level of education in Ghana has been discovered by other researchers such as Kirigia et al. (2005) and Mensah et al. (2010) as a significant factor for the people’s demand for insurance and medical care.

The marital status in general has no significant impact on membership, but single and widowed have higher subscription levels. It is argued that having children adds more responsibilities, and as such, puts parents/guardians with extra adversity to the jeopardy of health expenditures as compared to singles. This result contradicts (Kirigia et al., 2005, Harmon & Nolan, 2001; Liu & Chen, 2002), that married persons have a higher probability to buy health insurance over the single because they deemed it very significant to seek protection for their children.

The size of the family of the respondents was proved to have a statistically significant bearing with NHIS subscription. This could be attributed to the high subscription by married couples. It is believed that in order to not encounter any catastrophic health cost, people with high family size might demand health insurance more than those with smaller ones.

Respondents with high income stand a high chance of subscribing to the scheme. However, as their incomes grow bigger, they tend to look for other options elsewhere. This signifies the need for the scheme to revisit the benefit packages and the service provision to make it more attractive to high income earners. On the contrary, the results identify the problem of affordability as an impediment in the demand for insurance by the residents. This can be related to the general poverty headcount of the population, which is 28.5% (WB, 2013). This difficulty, relative to the current economic situation of the residents, calls for the re-examination of the exemption procedure of the scheme to benefit the poor and vulnerable in societies in order to realize the scheme slogan of “health insurance for all”. An economic intervention to improve the financial standings of the people must be fully endorsed by the authorities to enhance enrolment.

The results show a significant difference between membership and the place of residence. This again contradicts the findings by Gobah et al. (2011), that community description has no substantial impact in membership. The findings further produce enough evidence that the people who live in the rural areas have higher subscription (39.5%) than people in the urban centres (20.5%), a difference of 19%. This could be linked to the fact that people in the rural communities have limited access to health care services and needs to incur some cost to travel to the district capital or go beyond which is more costly to embark as compared to those in the cities.

The type of employment determines the amount one would receive at the end of the month. Findings showed that employment and the income level of the respondents have a significant effect on membership of NHIS subscription. This is in line with Kirigia et al. (2005), which depict that people who have secured jobs have a high chance to demand health insurance. The main occupation of the people in the municipality is agriculture (62.0%), which is predominantly on the subsistence basis. The income *vis-à-vis* expenditure patterns that suggest the bulk of their income goes to food (44.8%) followed by education (14.5%) before health (7.6%) (DDA, 2013). This informs policy makers to intensify education campaign on the need to invest more in their health and also support and encourage commercial farming to enrich income levels.

With regards to the reasons for not joining the NHIS, the majority of respondents stated that it was from financial difficulties; which is shown in figure 1. The financial problem raised by the majority of respondents was consistent with the results of Metiboba, 2011, which says that constrained in demanding health insurance was a result of several other factors in Nigeria, such as: poverty, poor supply of drugs or vaccines, inadequate trained health personnel, dwindling funding of health care, employers/providers’ resistance to contribute their quota, general poor state of the nation’s health care service, cultural belief systems and dilapidated health infrastructures. Also, WHO in 2003, highlighted the fact that membership rates in the health insurance scheme are often influenced by the size of the gap between the household’s home to the nearby health facility where covered services are delivered; which is in line with accessibility problem mentioned by respondents.

The suggestions made by the respondents, most importantly the reduction of the premium level, confirm the reason that the people have financial challenges affect their ability to demand NHIS.

## 5. Conclusion

Findings from the research suggest that residents’ decision to enrol is statistically significant with gender, education, number of children, place of residence, employment and financial constraints. Age and marital status of the respondents have no significant difference with NHIS subscription. It was also revealed that the most significant factor that has been the challenge for the people to join or not to join the NHIS is income. Concerns were raised by the respondents to improve the quality of service offered by health providers. Policy intervention that leads to a reduction in premium payment, ensuring that the enrolment campaign corresponds to the present financial sequences of the municipality, and assisting access to credit are all measures which is expected to increase accessibility; thus leading to greater membership rates. It is also significant to improve the quality of care to meet the needs and expectations of the general public. There is the need to provide a furnished medical facility to the doorsteps of the people to serve as a guarantee for easy access to health care services, reduction in time wasting and raise enough income to improve both manpower and infrastructure levels of facilities. NHIS must also consider an inclusion of herbal medicines into their drug list to attract those whose belief does not encourage the use of chemical drugs.

It must be noted that, the enrolment in the sample is higher than the national one, which gives good reasons to believe that an obstacle to enrolment goes beyond what was identified, which is a limitation of this study. It is recommended that similar research is conducted in a district which has low enrolment figures to find out the challenges affecting the demand of NHIS.
